# Full-Genome Sequence of a Reassortant H1N2 Influenza A Virus Isolated from Pigs in Brazil

**DOI:** 10.1128/genomeA.01319-14

**Published:** 2014-12-18

**Authors:** Candice Schmidt, Samuel Paulo Cibulski, Ana Paula Muterle Varela, Camila Mengue Scheffer, Adrieli Wendlant, Fabiana Quoos Mayer, Laura Lopes de Almeida, Ana Cláudia Franco, Paulo Michel Roehe

**Affiliations:** a Virology Laboratory, Department of Microbiology, Immunology and Parasitology, Institute of Basic Health Sciences, Federal University of Rio Grande do Sul (UFRGS), Porto Alegre, Rio Grande do Sul (RS), Brazil; b Fepagro Animal Health—Institute of Veterinary Research Desidério Finamor (IPVDF), Eldorado do Sul, Rio Grande do Sul, Brazil

## Abstract

In this study, the full-genome sequence of a reassortant H1N2 swine influenza virus is reported. The isolate has the hemagglutinin (HA) and neuraminidase (NA) genes from human lineage (H1-δ cluster and N2), and the internal genes (polymerase basic 1 [PB1], polymerase basic 2 [PB2], polymerase acidic [PA], nucleoprotein [NP], matrix [M], and nonstructural [NS]) are derived from human 2009 pandemic H1N1 (H1N1pdm09) virus.

## GENOME ANNOUNCEMENT

Influenza A viruses are important human and animal pathogens with a large impact on public and animal health. Swine influenza viruses (SIVs) of the H1N1, H1N2, and H3N2 subtypes remain endemic in swine populations worldwide (1). These contain eight RNA segments, of which the hemagglutinin (HA) and neuraminidase (NA) genes define the subtype. In case of a mixed infection by two viral strains within a cell, the exchange of gene segments may lead to the generation of a new reassortant virus. Due to this phenomenon, the origin of such viruses, as well as their genetic and antigenic characteristics, may vary in different geographic regions (2, 3).

Although SIV infections have already been detected in swine in Brazil, in most cases, these viruses have not had their full genomes sequenced. The complete genome sequences of two SIV isolates are reported here. The viruses were recovered from the lung tissues of piglets in an outbreak of respiratory disease that occurred in a certiﬁed pig breeder-producing farm in the state of Rio Grande do Sul, Brazil.

Virus isolation from lung tissues was performed according to standard procedures (4) in Madin-Darby canine kidney cells (MDCK). The presence of virus was confirmed with a reverse transcription-PCR (RT-PCR) targeting the matrix (M) gene of influenza A virus (5). Viral RNA was extracted from lung tissues using the TRIzol reagent (Life Technologies). Whole-genome sequences were generated by RT-PCR using the PathAmp FluA reagents (Life Technologies). The amplicons were purified with Agencourt AMPure XP-PCR purification (Beckman Coulter), followed by sequencing in the MiSeq sequencing platform (Illumina). The data were *de novo* assembled on BaseSpace Cloud (Illumina) by the SPAdes genome assembler (version 3.0) and analyzed by the Geneious software (version 7.1.7).

Genetic analysis revealed that the two isolates share 98.9 to 100% identity at the nucleotide level. The viral genomes of strains A/swine/Brazil/G2-P1/2013 (H1N2) and A/swine/Brazil/G2-P2/2013 (H1N2) consist of 8 single-stranded RNA segments: polymerase basic 2 (PB2), polymerase basic 1 (PB1), polymerase acidic (PA), hemagglutinin (HA), nucleoprotein (NP), neuraminidase (NA), matrix (M), and nonstructural protein (NS), with corresponding nucleotide lengths of 2,308, 2,341, 2,175, 1,746, 1,565, 1,465, 1,027, and 890. The encoded viral proteins and their amino acid lengths include PB2 (759), PB1 (757), PB1-F2 (91), PA (705), PA-X (241), HA (565), NP (498), NA (469), M1 (252), M2 (97), NS1 (219), and NS2 (121).

The amantadine resistance marker S31N (6) was observed in the M2 protein-coding region in the two genomes. Additionally, the PB1 gene encodes an infrequently occurring truncated protein, PB1-F2, of 91 amino acids (aa). Phylogenetic analyses reveal that the HA and NA genes clustered with influenza viruses of human lineage (H1-δ cluster and N2), whereas the internal genes (PB1, PB2, PA, NP, M, and NS) clustered with the A(H1N1) pdm09.

These results highlight the importance of continued surveillance of influenza A viruses, since the virus can recombine with viruses from different hosts, including pigs; such events may give rise to reassortants with significant pathogenic potential.

### Nucleotide sequence accession numbers.

The genome sequences of A/swine/Brazil/G2-P1/2013 (H1N2) and A/swine/Brazil/G2-P2/2013 (H1N2) are deposited in GenBank under accession numbers KP027582 to KP027589 and KP027590 to KP027597, respectively.
